# P-1608. No Targets, No Problem: Evaluation of Patients with Bloodstream Infections without a Molecular Target Detected by a Rapid Diagnostic Assay

**DOI:** 10.1093/ofid/ofae631.1775

**Published:** 2025-01-29

**Authors:** Megan Shah, Julie E Williamson, Lisa Davidson, Michael S Boger

**Affiliations:** Atrium Health, Charlotte, North Carolina; Atrium Health, Charlotte, North Carolina; Atrium Health, Charlotte, North Carolina; Atrium Health, Charlotte, North Carolina

## Abstract

**Background:**

Early identification and management of sepsis is essential in improving patient outcomes. Blood culture rapid diagnostic testing (RDT) allows for early targeting of antimicrobial therapy, particularly when paired with antimicrobial stewardship (AMS) intervention. Hospitalized patients with positive blood cultures are reviewed daily by AMS pharmacists in our health-system, regardless of the RDT result. Limited data exist on the impact of AMS assessment in patients with a positive blood culture with an organism that is not a target identified by RDT. The objective of this study is to describe the microbiology and antimicrobial management of patients with a positive blood culture, but no molecular target detected (NMTD) on the BioFire® Blood Culture Identification 2 (BCID2) panel.

**Figure:**
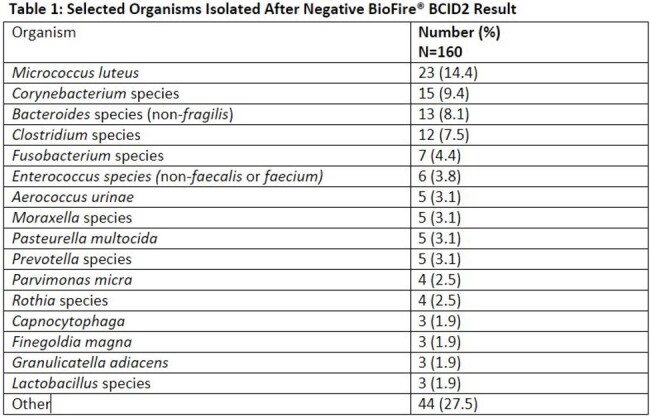

**Methods:**

This retrospective review evaluated patients within the Atrium Health Greater Charlotte Market who had ≥ 1 positive blood culture with a BCID2 result of NMTD from January to May of 2023. The primary endpoint was to identify the most common organisms recovered by standard of care methods following a NMTD result. Secondary endpoints were evaluated in hospitalized patients and included change in antimicrobials in response to the Gram stain result and percentage of patients on active therapy at organism identification and/or susceptibility testing. Data were analyzed using descriptive statistics.

**Results:**

160 organisms were identified in 149 patients with a BCID2 result of NMTD. The most common organisms identified were *Micrococcus luteus* (14.3%), *Corynebacterium* spp. (9.4%), non-*fragilis Bacteroides* spp. (8.1%), *Clostridium* spp. (7.5%), and *Fusobacterium* spp. (4.4%) (Table 1). Contamination was suspected in 34% of patients; however, when the organism was considered a true pathogen by the treating provider, the most common infection sources were intra-abdominal (26%) and skin/skin structure (12%). Antibiotics were modified after the Gram stain or NMTD results in 38% of patients, and 79/85 (93%) of patients were on active therapy at organism identification or susceptibility testing if considered a true infection.

**Conclusion:**

Even when RDT fails to yield a molecular target, AMS review of patients with positive blood cultures may ensure adequate antimicrobial therapy

**Disclosures:**

**All Authors**: No reported disclosures

